# Risk factors for hospital re-presentation among older adults following fragility fractures: protocol for a systematic review

**DOI:** 10.1186/s13643-015-0084-5

**Published:** 2015-07-11

**Authors:** Saira A. Mathew, Kristiann C. Heesch, Elise Gane, Steven M. McPhail

**Affiliations:** School of Public Health and Social Work and Institute of Health and Biomedical Innovation, Queensland University of Technology, Brisbane, Australia; Centre for Functioning and Health Research, Metro South Health, Queensland Department of Health, Brisbane, Australia; School of Health & Rehabilitation Sciences, The University Of Queensland, Brisbane, Australia

**Keywords:** Hospital readmissions, Fragility fractures, Geriatric, Older adults, Risk factors, Re-presentation

## Abstract

**Background:**

After being discharged from hospital following the acute management of a fragility fracture, older adults may re-present to hospital emergency departments in the post-discharge period. Early re-presentation to hospital, which includes hospital readmissions, and emergency department presentations without admission may be considered undesirable for individuals, hospital institutions and society. The identification of modifiable risk factors for hospital re-representation following initial fracture management may prove useful for informing policy or practice initiatives that seek to minimise the need for older adults to re-present to hospital early after they have been discharged from their initial inpatient care. The purpose of this systematic review is to identify correlates of hospital re-presentation in older patients who have been discharged from hospital following clinical management of fragility fractures.

**Methods/Design:**

The review will follow the PRISMA-P reporting guidelines for systematic reviews. Four electronic databases (PubMed, CINAHL, Embase, and Scopus) will be searched. A suite of search terms will identify peer-reviewed articles that have examined the correlates of hospital re-presentation in older adults (mean age of 65 years or older) who have been discharged from hospital following treatment for fragility fractures. The Effective Public Health Practice Project Quality Assessment Tool for Quantitative Studies will be used to assess the quality of the studies. The strength of evidence will be assessed through best evidence synthesis. Clinical and methodological heterogeneity across studies is likely to impede meta-analyses.

**Discussion:**

The best evidence synthesis will outline correlates of hospital re-presentations in this clinical group. This synthesis will take into account potential risks of bias for each study, while permitting inclusion of findings from a range of quantitative study designs. It is anticipated that findings from the review will be useful in identifying potentially modifiable risk factors that have relevance in policy, practice and research priorities to improve the management of patients with fragility fractures.

**Systematic review registration:**

PROSPERO CRD42015019379

## Background

The incidence of osteoporosis and frailty-related fractures is expected to rise as the number of older adults in the population increases [1, 2]. Osteoporosis is a silent metabolic bone disorder that causes bones to become weak and brittle. As the condition lacks any overt symptoms, it is often undiagnosed and untreated until a fragility fracture occurs [3]. Fragility fractures are fractures sustained from relatively minor forces (e.g. a fall from a standing height or less) and often occur among people with osteoporosis [4]. These fractures typically occur in vertebrae, femurs, distal forearms or proximal humeri [5], and they are associated with increased risk of disability, hospitalisation and mortality [6]. Fracture-related mortality among older adults may or may not occur within close temporal proximity to the fracture event. Premature death may occur as the end point in sequelae of negative health events precipitated by a fragility fracture months or years earlier [3].

The management of fragility fractures requires substantial investment from healthcare services, including provision of emergency department (ED) resources and inpatient hospital admissions [7–9]. The acute orthopaedic management of fragility fractures ensures that the fracture is adequately aligned and stable and may include surgical interventions. Ongoing recovery and rehabilitation may require a long period of time and care from multi-disciplinary teams [10, 11]. When older adults with fragility fractures are discharged from the hospital, they may develop complications directly related to the fracture management (e.g. surgical site infections) [12]. They may also experience complications associated with the fracture but also attributable, at least in part, to underlying comorbidities and low physical reserve (e.g. cardiovascular or respiratory complications) [12–14]. Frail older adults are at higher risk of adverse outcomes than younger adults who experience similar injuries [15, 16].

Age-related frailty develops as a consequence of decline in physiological systems and the subsequent age-related loss in physical, cognitive, social and psychological functioning [15, 6]. The degree of frailty experienced by older adults varies considerably, depending on clinical, personal and environmental factors. These factors can also increase the risk of requiring a re-presentation to hospital. Hospital re-presentations are visits to the hospital emergency department with or without inpatient admissions [17, 18]. They comprise ED visits without admission, same-day discharges and inpatient admissions for one or more days [18]. Re-presentation rates within 3 months of hospital discharge in older adults have been reported to vary between 16 and 19 % [19]. These rates typically include re-presentations due to conditions present or associated with the initial hospitalisation, fracture non-union, mal-union, re-fracture at the same site or new fractures elsewhere [3, 19, 20]. Appropriate clinical care during and following the initial hospitalisation may reduce subsequent hospital re-presentations [21, 22].

Re-presentations to hospital following fragility fractures may pose a substantial burden for individuals, hospital institutions and society. For individuals, the outcomes associated with re-presentation to hospital may include reduced physical and psychological wellbeing, function, health-related quality of life and increased risk of mortality [21, 23]. For hospital institutions, higher re-presentation rates may be seen as indicators of poor hospital performance and influence financial reimbursements for clinical care [24]. From a societal perspective, hospital re-presentations are a substantial economic burden due to the additional resources required for direct healthcare provision, as well as a reduced availability of healthcare resources for delivering care to other patients.

Studies investigating the correlates of hospital re-presentations, most notably readmissions to a hospital, have been conducted. However, there has not yet been a systematic review to identify correlates of re-presentations among older adults who have been hospitalised following fragility fractures. This protocol describes a systematic review that will examine the correlates of hospital re-presentation among older adults with fragility fractures.

### Objectives

Older adults who are discharged from hospital following initial management of fragility fracture may subsequently re-present to the hospital. The objective of this review is to identify patient, clinical or hospital-related factors that may be associated with hospital re-presentations in older adults following fragility fractures. The systematic review will synthesise existing knowledge and make recommendations for future research.

## Methods/Design

### Design

This systematic review will follow the Preferred Reporting Items for Systematic Reviews and Meta-Analyses Protocol (PRISMA-P) statement [25]. The PRISMA-P consists of a 17-item checklist intended to facilitate the preparation and reporting of protocols for a systematic review.

### Inclusion criteria

Studies will be selected according to the criteria outlined below. In summary, studies among older adults that have identified correlates of hospital re-presentations following an initial hospitalisation for management of a fragility fracture will be included.

#### Types of studies

Quantitative studies published in peer-reviewed journals will be included in this review. The systematic review will consist of studies using either epidemiological or experimental designs where analyses for potential correlates of hospital re-presentations have been reported. Studies will most likely be retrospective or prospective longitudinal cohort studies. However, analysis of factors associated with re-presentation to hospital from studies using other quantitative study designs (e.g. randomised controlled trials or non-randomised trials) will also be included. Qualitative studies, case studies and abstract-only reports, for which full text is not available, will be excluded from this review. Grey literature, including government reports, book chapters, theses and dissertations, will also be excluded.

#### Participants

Studies that sample older adults with fragility fractures will be included with no sex, race, ethnicity, residential status (patients living in residential care facilities, or elsewhere in the community will be included) or socioeconomic status restrictions. The terms “elderly” and “older adults” have not been defined consistently across the literature. For the purpose of this review, findings from studies of older adults (mean age 65 years or older) will be included. Sixty-five years of age was selected as the minimum age limiter as it is both widely used in scholarly literature in the field and is available as an age limiter in the CINAHL, PubMed and Embase database search functions [26–28]. Those studies that examine the predictors of re-presentations for older adults across multiple disease conditions but have separate statistical analysis reported for re-presentations in older adults (65 years and over) with fragility fracture will also be included.

#### Outcomes

Correlates of hospital re-presentations are the primary outcomes for this review. Re-presentation-related outcomes of secondary interest to the study are the number and frequency of re-presentations, reasons for re-presentation, rates of re-presentation and days since discharge to re-presentation. It is anticipated that re-presentations to hospital are most likely to occur between 28 days and 6 months following discharge [20], but it is plausible that re-presentation may occur outside this time frame. Therefore, this review will include studies that report re-presentations without any prescriptive restriction on the reason for representation anytime within the 2 years following initial hospitalisation for management of a fragility fracture.

### Search strategy

The literature will be searched using medical subject headings (MeSH) and combinations of key terms in four online databases. The search is limited to English language publications with no restriction on the year of publication. The electronic databases searches will include Embase (1945–present), PubMed/MEDLINE (1966–present), Scopus (1960–present) and CINAHL via EBSCO interface (1989–present) and use key terms contained in the title, abstract and keywords. In addition, the reference list of articles meeting the criteria for this review will be searched for further relevant studies.

The search syntax to be used across the databases is displayed in Table 1 and reflects the minor variation in search functionality across the respective databases. Search strings will focus on the terms readmission, fracture, elderly, hospital and their synonyms and will be combined with Boolean operators to produce the most relevant results. Age (>65 years) will be used as a limiter in all databases except Scopus, which does not have an age limiter function. Age limiter “age >65 years” captures studies with older adults 65 years and over.

**Table 1:** Search syntaxes

Database	Search syntax
PubMed	(fracture[MeSH Terms]) AND (((readmi* or rehosp* or re-admi* or re-hosp* or re-presentation)) OR "Patient Readmission"[Mesh]) Filters: Aged: 65+ years
CINAHL	"fracture* AND ( readmi* or rehosp* or re-admi* or re-hosp* or re-presentation) Age Groups: Aged: 65+ year
Embase	'fracture'/exp and ( readmi* or rehosp* or re-admission or re-hospitalisation or re-hospitalisation or re-presentation) AND ([aged]/lim OR [very elderly]/lim)
Scopus	ABS fracture* AND ( readmi* OR rehosp* OR re-admission OR re-hospitalisation OR re-hospitalisation or re-presentation) AND ( aged OR elderly OR geriatric OR old* )

### Data collection and analysis

#### Selection process

The literature will be searched by one member of the research team (SAM), and results will be imported into reference management software where duplicate references will be deleted. The selection process will consist of three stages that will be conducted by two authors (SAM, EG) independently of each other. The first and second stages will include screening titles then abstracts, respectively, against the selection criteria. Lastly, remaining articles will have their full texts examined against the selection criteria to arrive at a final list of articles to be included in the review. A third author (SMM) will arbitrate any unresolved disagreements arising during any stage in the selection process. A PRISMA flow diagram will be used to report the number of studies that are included and excluded in each stage of the selection process. This will include the number of articles that are carried forward to the next stage in the selection process as well as the number of articles excluded (and reason for exclusion), as recommended by the PRISMA-P statement.

#### Data extraction

Quantitative data from papers included in the review will be extracted and reported in a tabulated format. The data to be extracted will include details about samples, patient characteristics, study methods, inclusion and exclusion criteria, risk factors, statistical analysis and outcomes of importance to the review question and specific objectives (Table 2). Further data will be considered if available. Data extraction will be conducted independently by SAM and EG. SMM will arbitrate unresolved disagreements regarding data extraction.

**Table 2:** Data extraction variables

Content	Variable(s)
General study information	• Author
	• Date of publication
	• Country
	• Study time period
	• Other
Study design	• Prospective or retrospective cohort or RCT, non-randomised trial
Inclusion and exclusion criteria	• Population
	• Sample size
	• Patient characteristics (e.g. age, gender)
	• Disease characteristics
Risk factors/Correlates	• Patient factors (comorbidities)
	• Clinical (surgical, ASA score, medications)
	• Hospital (tertiary/secondary, LOS, admission type, reason for initial hospital visit, ward type)
	• Other
Primary outcome	• Re-presentation details
	• Number of re-presentations
	• Reason for re-presentations
	• Re-presentation rate
	• Time of re-presentations (time between initial hospital visit and re-presentation )
	• Other
Patient outcome	• Complications
	• Mortality
	• Functional independence (FIM)
	• Health-related quality of life (HRQOL)
Statistical analysis	• Analysis conducted
	• Results reported

#### Quality of the evidence

The quality of individual studies and risk of bias will be assessed using the Effective Public Health Practice Project Quality Assessment Tool [29]. This quality assessment tool will allow the review team to rate the relevant methodological parameters of studies across six areas: selection bias (selection of target population), study design (type of study), confounders (whether confounders were controlled for in the study design or analysis), blinding (patients’ or researchers’ awareness of an intervention, if applicable), data collection methods (validity and reliability) and withdrawals and dropouts (dropout rates and completion of study rates). The quality of the studies will be rated according to the global rating of the assessment tool as strong, moderate or low. Quality appraisals will also be conducted independently by SAM and EG, with SMM to arbitrate unresolved disagreement in quality ratings.

#### Data synthesis and reporting

The correlates of hospital re-presentation that are outlined in the included reports will be summarised. A best evidence synthesis [30, 31] will be implemented to integrate the strength of evidence of studies. This approach includes consideration of the methodological quality of studies and the consistency of results across studies. The review will also report the strengths and limitations of individual studies. Recommendations for future research and implications for preventing re-presentations to hospital will be discussed.

The authors consider it likely that clinical or statistical heterogeneity will prohibit valid meta-analyses from being conducted. However, if studies are found to be sufficiently homogenous and rigorous, a meta-analysis will be conducted. Clinical heterogeneity will be assessed using the information on each study’s sample, study setting and interventions received (as applicable). Methodological heterogeneity will be assessed using data extracted on study design, procedures and correlates reported. Suitability for pooling and selection of a meta-analytic model (e.g. random effects) will be determined after consideration of clinical and methodological homogeneity. It is anticipated that the primary outcome of interest (re-presentation to hospital) will be reported and analysed in the primary studies as a categorical variable and if a meta-analysis is able to be conducted, use of relative risk (RR) will be the appropriate indicator of effect for potential correlates in this review. However, it is plausible that other measurement and analytical approaches will be reported in the primary studies and thus alternative meta-analytical approaches (e.g. use of correlation coefficients or hazard ratios) will be used. Forest plots will be generated to present pooled coefficient estimates derived from studies included in meta-analyses. *I*^2^ values will be used as a measure of the level of consistency across studies, with 0, 25, 50 and 75 % considered no, low, moderate and high levels of heterogeneity, respectively [32] Examination of potential moderator effects and sensitivity analyses will also be considered if findings and methodological rigour of analysed studies allow for these analyses. Exploration of findings for body region subgroups will be considered where numerous studies exist for the same body region.

## Discussion

This review will systematically retrieve and examine studies reporting potential correlates of re-presentation to hospital following fragility fractures among older adults. Although clinical and methodological heterogeneity across studies may impede meta-analyses, the best evidence synthesis will outline correlates of hospital re-presentations in this clinical group. This synthesis will take into account potential risks of bias for each study, while permitting inclusion of findings from a range of quantitative study designs. It is anticipated that findings from the review will be useful for informing policy, practice and research priorities for improving the management of patients with fragility fractures who present to the hospital.

## Abbreviations

ED: emergency department
LOS: length of stay
RCT: randomised controlled trials
ASA: American Society of Anaesthesiologists Score
ABS: abstract
